# Signaling in the Tomato Immunity against *Fusarium oxysporum*

**DOI:** 10.3390/molecules26071818

**Published:** 2021-03-24

**Authors:** Francisco Hernández-Aparicio, Purificación Lisón, Ismael Rodrigo, José María Bellés, M. Pilar López-Gresa

**Affiliations:** Instituto de Biología Molecular y Celular de Plantas, Universitat Politècnica de València—Consejo Superior de Investigaciones Científicas, 46022 Valencia, Spain; Francisco-Jorge.HERNANDEZ@danone.com (F.H.-A.); plison@ibmcp.upv.es (P.L.); irodrig@ibmcp.upv.es (I.R.); jmbelles@btc.upv.es (J.M.B.)

**Keywords:** *Fusarium*, tomato, natural products, volatiles, biotic interaction, metabolomics, GC-MS

## Abstract

New strategies of control need to be developed with the aim of economic and environmental sustainability in plant and crop protection. Metabolomics is an excellent platform for both understanding the complex plant–pathogen interactions and unraveling new chemical control strategies. GC-MS-based metabolomics, along with a phytohormone analysis of a compatible and incompatible interaction between tomato plants and *Fusarium oxysporum* f. sp. *lycopersici,* revealed the specific volatile chemical composition and the plant signals associated with them. The susceptible tomato plants were characterized by the over-emission of methyl- and ethyl-salicylate as well as some fatty acid derivatives, along with an activation of salicylic acid and abscisic acid signaling. In contrast, terpenoids, benzenoids, and 2-ethylhexanoic acid were differentially emitted by plants undergoing an incompatible interaction, together with the activation of the jasmonic acid (JA) pathway. In accordance with this response, a higher expression of several genes participating in the biosynthesis of these volatiles, such as *MTS1*, *TomloxC,* *TomloxD,* and *AOS*, as well as *JAZ7*, a JA marker gene, was found to be induced by the fungus in these resistant plants. The characterized metabolome of the immune tomato plants could lead to the development of new resistance inducers against *Fusarium* wilt treatment.

## 1. Introduction

Tomatoes (*Solanum lycopersicum*) are an important crop, whose cultivation and consumption are both constantly increasing, reaching a worldwide production of more than 180 million tons on a cultivated area of almost 4.8 million hectares in 2018 [1]. In addition, the tomato is an excellent model plant for studying plant–pathogen interactions, and the results of such research can be scaled-up to other ones. For its economic importance worldwide, the control of main pests or diseases becomes essential to prevent yield and productivity losses in tomato crops. Among the most common tomato diseases, there are those caused by bacteria such as *Pseudomonas syringae*, viruses like tomato mosaic virus (ToMV), and pathogenic fungi such as *Botrytis cinerea, Phytophthora infestans,* and *Fusarium oxysporum.* Specifically, *F. oxysporum* f. sp. *lycopersici* (*Fol*) is the forma specialis that affects tomato plants [2] and represents the fifth most important plant pathogenic fungus around the world responsible for economic losses around 14% in tomato crops [3].

*Fol* has hemibiotrophic behavior, living in soil during long periods before infecting a new host. The pathogenic isolates are able to reach the central cylinder of the roots through centripetal intracellular growth by digestion of the cell wall, mainly via intercellular progress [4]. Once *Fol* reaches the xylem, its growth within the xylem vessels goes through the hypocotyl and the petioles. This mycelia growth and the defensive system of the plant bring about a progressive obstruction of the vessels and the consecutive collapse of the water and nutrient supply. In tomato plants, *Fol* causes a yellowing and withering of the upper leaves, browning of the hypocotyl vessels, and death in long term infection [5].

The use of fungicides against fusarium wilt during cropping is ineffective, and therefore prevention is necessary. Besides, due to its persistence in soil and its easy dissemination through seeds and wind, fusarium wilt is difficult to control, with the possibility of scaling up as a consequence of global warming. Nowadays, the most effective means of control is the use of resistant cultivars, but the co-evolution of resistant genes and different formae speciales makes this interaction very complex [6]. There are eleven candidates for effectors or avirulence genes in *Fol*, denominated SIX (secreted in xylem), which contribute to the pathogen virulence [7]. Among them, *Avr1*, *Avr2*, and *Avr3* are widely described [8]. Tomato plants containing the *I* (immunity) resistance genes (*I*, *I2*, *I3* and *I7*) become resistant to fungal infection after the corresponding *Avr* recognition, therefore activating the effector-triggered immunity (ETI), which is characterized by the development of the so-called systemic acquired resistance (SAR), and resulting in an incompatible interaction [9]. The complexity of this interaction lays on the suppression by *Avr1* of *I2* and *I3* mediated resistance in tomato plants [8]. Besides, there is no further knowledge about the pathogenic action of the other SIX effectors [10]. In contrast, tomato plants deficient in the *I* resistance genes are susceptible to fungal infection by a lack of “gene for gene” recognition, resulting in a compatible interaction.

Different phytohormones such as salicylic acid (SA), jasmonic acid (JA), abscisic acid (ABA), and ethylene (ET) have been involved in tomato plant response against different pathogens [11,12]. However, the contribution of these signal molecules in both compatible and incompatible tomato–*Fol* interaction has not yet been explored.

Metabolomics has emerged as a powerful platform for both understanding the complex plant–pathogen interactions and unraveling new strategies for chemical control. Non-targeted metabolomics approaches based on nuclear magnetic resonance (NMR) or mass spectrometry (MS) have been applied to provide rapid and accurate information about the defense metabolites implicated in different pathogen–tomato interactions such as tomato–citrus exocortis viroid (CEVd) [13], tomato–*Pseudomonas syringae* [14], tomato–ToMV [15], or tomato–*Phytophtora infestans* [16]. Specifically, the application of MS techniques coupled with gas (GC) or liquid chromatography (LC) have allowed for the identification of differentially emitted volatile organic compounds (VOCs) or accumulated metabolites in the resistant tomato cultivars to create new chemical control strategies.

In the present article, an untargeted GC-MS metabolomics study of the compatible and incompatible interaction between the *Fol* race 1 and isogenic lines of tomato plants, either containing or not containing the corresponding *I* resistance gene, was carried out to identify the chemical composition of the aroma emitted by the resistant tomato plants. Moreover, the induction of some genes involved in the VOC biosynthesis pathways and the activation of the main signaling defensive routes along with the levels of SA, JA and ABA were analysed in both interactions. The obtained results highlight the specific defensive phytohormones and VOCs involved in the immune tomato response against *Fol*.

## 2. Results

### 2.1. Movione Tomato Plants Harbouring the I Resistance Gene Are Immune to Fol Infection

**Figure 1:**
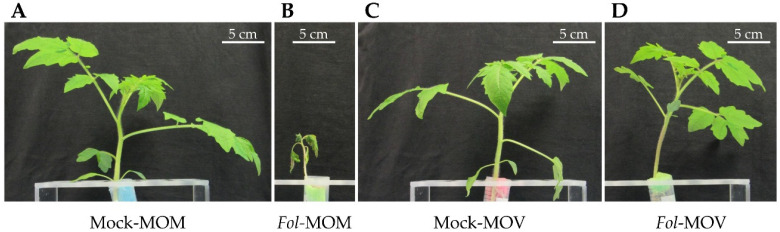
Fusarium wilt symptoms in Momor (MOM) and Movione (MOV) tomato plants at 14 dpi after *Fusarium oxysporum* f. sp. *lycopersici* (*Fol*) inoculation. Tomato plants display the representative phenotype observed in infected susceptible MOM (**B**) compared to mock-inoculated (**A**), and infection-resistant MOV (**D**), as compared to their corresponding mock-inoculated (**C**).

To characterize the establishment of the ETI, a symptomatology analysis of *Fol*-inoculated Movione (MOV) tomato plants carrying the *I* resistance gene was performed. Concurrently, Momor (MOM) tomato plants lacking the *I* resistance gene were used to compare the fusarium wilt symptoms. As Figure 1 shows, evident differences in symptom severity were observed between both isogenic lines at 14 days post-inoculation (dpi).

As Figure 1C,D show, MOV displayed no phenotypical differences between mock- and *Fol*-inoculated plants, confirming the establishment of ETI. On the contrary, *Fol*-infected MOM plants were characterized by a height and weight reduction, yellowing of the leaves, browning of the xylem vessels, and even a total collapse of most plants, therefore confirming their susceptibility to *Fol* (Figure 1B). Finally, no differences between both non-inoculated isogenic lines were observed (Figure 1A,C).

To better quantify the differences between infected MOV and MOM plants, several morphological parameters were measured such as height, weight, the ratio between the hypocotyl length and the epicotyl length in reference to the height, as well as the water consumption per plant (Table S1, Supplementary Materials). Infected tomato plants were classified according to their disease index [17] as a medium value out of the score of the symptoms from mild (1) to very severe (4) along the infection (see Materials and Methods).

As expected, a statistically significant reduction of height, weight, hypocotyl elongation, and symptom severity was observed between both isogenic lines, confirming the resistance of MOV tomato plants to *Fol* infection.

To study the possible relationship between the observed symptoms and the presence of the pathogen, *Fol* levels were analyzed in the hypocotyl of both infected isogenic lines at 7 and 14 dpi by qPCR, being statistically higher in MOM than in MOV plants at any time (Figure S1), and correlating with the symptoms observed in Figure 1.

### 2.2. Movione Tomato Plants Activate an Early JA-and ABA-Mediated Defense Response after Fol Infection

**Figure 2:**
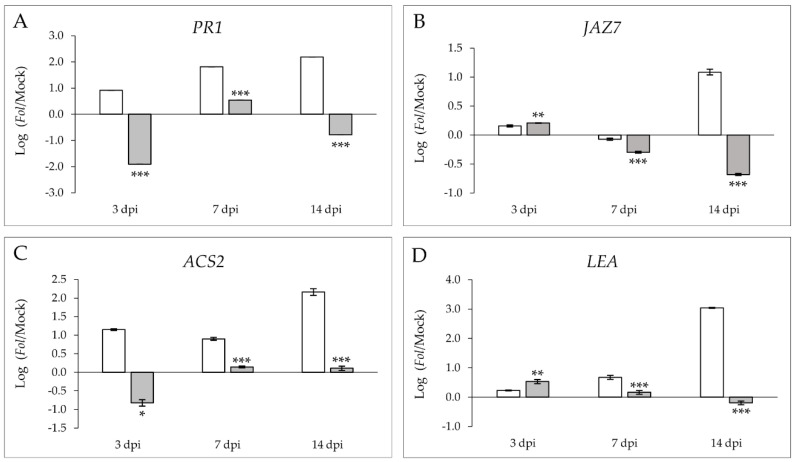
Logarithm of the ratios of the relative expression levels of *PR1* (**A**), *JAZ7* (**B**), *ACS2* (**C**), and *LEA* (**D**) between *Fol*-inoculated and mock-inoculated susceptible Momor (MOM, white) and resistant Movione (MOV, gray) tomato plants upon *Fusarium oxysporum* f. sp. *lycopersici* infection at 3, 7, and 14 dpi by RT-qPCR. Values obtained were normalized in relation to the elongation factor 1 α (accession X53043.1). cDNA expression levels were expressed as the average log(ratio) values of a representative experiment. Asterisks (*) point out statistical differences between mock-inoculated and *Fol*-inoculated MOM and MOV plants according to *t*-test or Mann–Whitney test, with *p* < 0.05 (*), *p* < 0.01 (**), and *p* < 0.001 (***).

To better characterize the infection in both isogenic lines, the activation of the main signaling defensive routes was studied. The expression levels of different marker genes corresponding to the pathways mediated by SA, JA, ET, or ABA, which are the most important phytohormones involved in plant defense, were analyzed by using RT-qPCR (Figure 2) both in susceptible Momor (MOM) and resistant Movione (MOV) tomato plants after 3, 7, and 14 days of *Fol* inoculation. To avoid possible differences between the Moneymaker isogenic lines, logarithm of the ratios (log(ratio)) of the relative expression levels between *Fol*-infected and mock-inoculated plants were calculated; log(ratio) above 0 indicating a *Fol*-provoked induction of the marker gene.

The expression of *PR1* (pathogenesis-related 1), which is the marker gene for SA-mediated plant response [18], showed a statistically significant increase in the log(ratio) of the susceptible isogenic line when compared to the resistant plants, in accordance with the symptom development (Figure 2A). Specifically, the log(ratio) of the induction of *Fol*-infected MOM plants with respect to the mock MOM plants was 1-, 1.8-, and 2.2-fold at 3 dpi, 7 dpi, and 14 dpi, respectively. In contrast, MOV plants showed a log(ratio) below 0 at 3 dpi (−1.9) and 14 dpi (−0.8), thus indicating a *Fol*-mediated downregulation of *PR1* in this resistant isogenic line.

The analysis of *JAZ7* (jasmonate ZIM-domain 7) (Figure 2B), a gene related to the JA pathway [19], showed similar results at the end of the fungal infection, while a statistically significant increase in the log(ratio) was found at 3 dpi (0.2) in the resistant MOV plants with respect to the susceptible MOM.

*ACS2* (aminocyclopropane-1-carboxylate synthase 2) expression was chosen to follow the ethylene-mediated signalling [20]. No relevant induction was found in the resistant MOV tomato plants upon fungal inoculation at any time point since the log(ratio) was negative (–0.8 at 3 dpi) or around 0 (0.1 at both 7 and 14 dpi) (Figure 2C). However, *ACS2* was significantly induced in the *Fol*-infected MOM plants during the pathogenic interaction, displaying a log(ratio) of 1.2, 0.9, and 2.2 at 3, 7, and 14 dpi, respectively.

Differences between the log(ratio) of MOM and MOV plants were observed for *LEA* (late embryogenesis abundant) (Figure 2D), a gene related to ABA [21], with these differences being statistically significant at any time point. It should be noted that there was a significant induction of *LEA* (0.5) at 3 dpi in the resistant MOV plants compared to the susceptible MOM. Nevertheless, a decrease in the expression pattern for these genes was found in MOV plants along the infection process.

Our results suggest that the susceptible MOM plants activated all the main defense pathways along the infection, this response being late and insufficient to stop the disease progression in accordance with the symptom development. However, MOV plants were characterized by an early induction of the JA and ABA pathways, with an antagonistic effect on the SA and ET mediated response at 3 dpi, suggesting that the JA-and ABA-mediated responses could be effective enough to prevent the symptom development and to establish ETI.

### 2.3. Levels of Salicylic Acid and Abcisic Acid Are Enhanced in the Susceptible MOM Tomato Plants Infected by Fol

**Figure 3:**
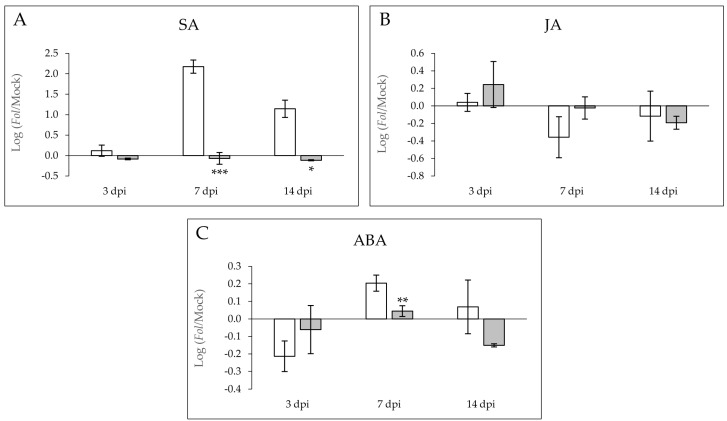
Logarithm of the ratios of the SA (**A**), JA (**B**) and ABA (**C**) phytohormone levels in susceptible Momor (MOM; white) and resistant Movione (MOV; gray) tomato plants upon *Fusarium oxysporum* f. sp. *lycopersici* inoculation at 3, 7 and 14 dpi, with respect to the corresponding mock-inoculated plants. Values were expressed as the average log(ratio) of data corresponding to a representative experiment. Asterisks (*) point out statistical differences according to *t*-test or a Mann–Whitney test, with *p* < 0.05 (*), *p* < 0.01 (**) and *p* < 0.001 (***).

Levels of the main defense phytohormones SA, ABA, and JA were analyzed in the MOM (susceptible) and MOV (resistant) tomato plants infected with *Fol* in a time-course study. Figure 3 represents the log(ratio) of the phytohormone accumulation between *Fol*-infected and mock-inoculated plants in both MOM and MOV isogenic lines.

A statistically significant accumulation of SA (Figure 3A) and ABA (Figure 3C) was observed in the compatible interaction at 7 dpi. At this time point, the SA and ABA log(ratio) in susceptible tomato plants was around 2.2 and 0.2, respectively, indicating that these phytohormones are involved in the plant response against *Fol*. Besides, SA levels were significantly higher in the susceptible plants at the end of the fungal infection (14 dpi). According to the higher expression of *LEA* observed at 3 dpi (Figure 2D), a lower reduction of ABA levels was detected in resistant MOV plants at that time point. In contrast, an inverted pattern was detected for JA (Figure 3B) in this interaction, observing a trend of JA accumulation at 3 dpi in the resistant MOV plants when compared to susceptible MOM. Although no statistical differences were detected in JA levels due to the high variability displayed, these results entirely agree with the *JAZ7* induction (Figure 2B), therefore reinforcing the idea that an early activation of the JA pathway occurs in the resistant plants. On the contrary, SA accumulation is a distinctive feature of the susceptibility to *Fol*, perfectly correlating with the *PR1* expression.

### 2.4. Susceptible MOM and Resistant MOV Tomato Plants Display Different Volatile Chemical Composition upon Fol Infection

**Figure 4:**
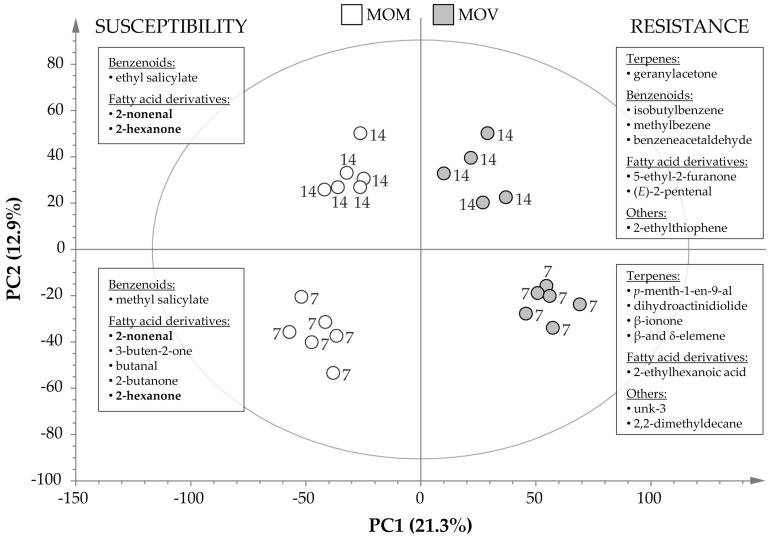
Score plot of the PCA based on the whole array of the mass spectra within an *m*/*z* range from 35 to 250. VOCs were analyzed from susceptible Momor (MOM; white) and resistant Movione (MOV; gray) tomato plants infected by *Fusarium oxysporum* f. sp. *lycopersici* at 7 and 14 dpi. PC1 and PC2 explain the 19% and 12% of variance separating the samples according to the type of interaction and timing of the fungal infection, respectively. Statistically differential metabolites according to *t*-test for both incompatible (MOV/MOM ratio ≥ 1.7) and compatible (MOV/MOM ratio ≤ 0.8) interactions are shown in boxes.

To identify the VOCs involved in the establishment of ETI, changes in the levels of these metabolites in mock-inoculated and *Fol*-infected MOV and MOM tomato leaves were analyzed by GC-MS at 7 and 14 dpi. Based on the massive dataset obtained (11,505 detected peaks) from the chromatographic study, a principal component analysis (PCA) was performed. The first analysis revealed that both mock-inoculated isogenic lines constitutively emitted a different profile of VOCs (Figure S2). These results could be due to the differences among both isogenic lines caused by the integration of the *I* resistance gene in MOV plants. Consequently, the data set was normalized by the mean of the height ions from each mock-inoculated isogenic line to eliminate this metabolic difference, and a new analysis containing the normalized infected plants was performed (Figure 4). The score plot of PCA clearly showed that the first component (PC1) explained changes in the chemical composition of the susceptible and resistant infected tomato plants (21% of variance), while the metabolic alteration during the time course of the experiment was clearly distinguished by the second component (PC2, 13% of variance). The analysis supported that different metabolites seem to be involved in the compatible and incompatible interaction and depending on time of infection. Analyzing the PC1 of the loading plot, the specific VOCs that strongly contributed to the separation of samples according to the interaction type were identified.

The statistical volatiles differentially released during ETI establishment in resistant MOV tomato plants infected by *Fol* were extracted from the positive part of PC1 and are listed in Table S2 (MOV/MOM ratio > 1). Among them, some terpenoids were significantly emitted by resistant plants after 7 days of fungal inoculation: the monoterpene 3-carene, along with two aldehydic monoterpenoids and (*Z*)-linalool oxide, two nor-isoprenoids (dihydroactinidiolide and β-ionone), and four sesquiterpenes (α-caryophyllene and three isoforms of elemene). In addition, 2-ethylhexanoic acid, 2,2-dimethyldecane, and three unknown compounds (unk 1–3) were the volatiles significantly over-emitted by the resistant tomato plants at the beginning of the infection. Interestingly, the volatile profile at 14 dpi was characterized by the emission of benzenoids and C5-fatty acid derivatives such as isobutylbenzene, benzeneacetaldehyde, methylbenzene, 5-ethyl-2-furanone, and (*E*)-2-pentenal. Only the sesquiterpenes α- and β-caryophyllene were also significantly induced in the incompatible interaction together with the apocarotenoid geranylacetone, an unknown nitrogen compound (unk 2), the hydrocarbon 2,2-dimethyldecane, and the sulfur compound 2-ethylthiophene (Figure 4). Particularly, α-caryophyllene, 2,2-dimethyldecane, and unk 2 were significantly over-emitted by MOV plants during all of the experiment.

Similarly, the analysis of the negative side of the PC1 loading plot allowed for identifying the VOCs emitted by the susceptible MOM tomato plants upon *Fol* infection, showing the metabolites detailed in Table S3 (MOV/MOM ratio < 1) and summarized in Figure 4. Benzenoids and fatty acid derivatives were differentially over-emitted by the vulnerable plants. In the first group, methyl salicylate (MeSA) and ethyl salicylate (EtSA) were over-emitted at 7 and 14 dpi, respectively. On the other hand, the production of some aldehydes and ketones derived from the fatty acid degradation as 2-nonenal, 2-hexanone, 2-heptanone, and 3-heptanone was induced during all of the infection process. However, 3-buten-2-one, butanal, and 2-butanone were emitted exclusively at the beginning of the infection process, and 2-pentanone at the end. The statistically significant and most over-emitted discriminant VOCs from the resistant MOV (MOV/MOM ratio ≥ 1.7) and the susceptible MOM (MOV/MOM ratio ≤ 0.8) tomato plants are summarized in Figure 4.

### 2.5. Fungal Infection Induces the Specific Expression of Genes Involved in VOC Biosynthesis

**Figure 5:**
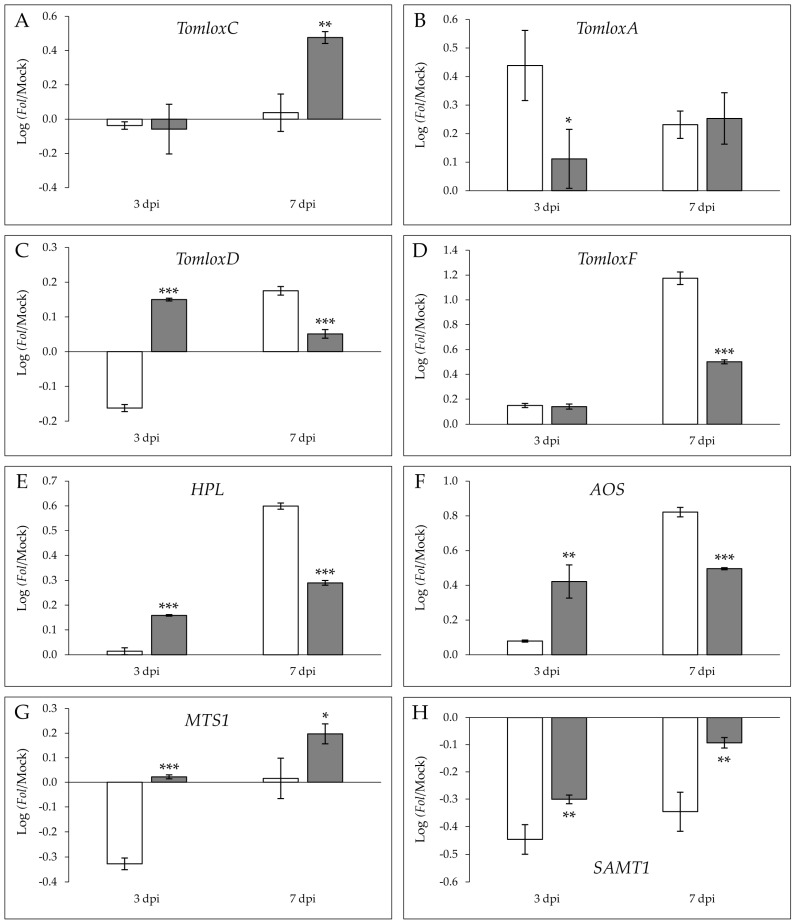
Logarithm of the ratios of the relative expression levels between *Fol*-infected and mock-inoculated leaves of *TomloxC* (**A**), *TomloxA* (**B**), *TomloxD* (**C**), *TomloxF* (**D**), *HPL* (**E**), *AOS* (**F**), *MTS1* (**G**), and *SAMT1* (**H**) in susceptible Momor (MOM; white) and resistant Movione (MOV; gray) tomato plants upon *Fusarium oxysporum* f. sp. *lycopersici* infection at 3 dpi and 7 dpi by RT-qPCR. cDNA expression levels were expressed as the average log(ratio) values of a representative experiment. Values were expressed in relation to the endogenous gene, elongation factor 1 α. Statistical analysis was done by means of a *t*-test or a Mann–Whitney test. Asterisks (*) mean *p* < 0.05 (*), *p* < 0.01 (**), and *p* < 0.001 (***).

To relate the differential volatile production with the transcriptional activation, the expression levels of several key genes involved in VOC biosynthesis were analyzed by RT-qPCR (Figure 5). To study the genes involved in aldehydes, esters, and alcohols of 9-, 6-, and 5-carbons, and jasmonate biosynthesis, the possible activation pathway of fatty acid derivatives was analyzed. In this sense, the lipoxygenases (LOX) are a group of enzymes responsible for converting both linoleic and linolenic acids into 9- and 13-hydroperoxides which are cleaved by hydroperoxidelyases (HPL) to form the corresponding short-chain aldehydes and oxo-acids. Besides, the allene oxide synthase (AOS) produces jasmonate derivatives from 13-hydroperoxide [22]. Six genes which codify for lipoxygenases were described in tomatoes [23]. *TomloxA*, *TomloxB,* and *TomloxE* codify for 9-LOX, which catalyzes the first step in the synthesis of 9C compounds, while *TomloxC*, *TomloxD,* and *TomloxF* codify for 13-LOX involved in the formation of oxylipins. Specifically, *TomloxD* is responsible for JA emission in the wound response, and *TomloxC* and *TomloxF* take part in the production of 5-carbon and 6-carbon volatiles, respectively. In addition to genes involved in fatty acid derivatives, *MTS1* and *SAMT1* were studied as marker genes for the biosynthesis pathways of terpenoids like monoterpenes and sesquiterpenes, and benzenoids, respectively [22]. Log(ratio) of the relative expression levels of these genes between *Fol*-infected and mock-inoculated plants in both MOM and MOV isogenic lines is represented in Figure 5.

A significant induction of the relative expression levels was observed for *TomloxA* at 3 dpi (Figure 5B), as well as for *TomloxF* (Figure 5D) and HPL (Figure 5A) at 7 dpi in susceptible MOM with respect to resistant MOV plants, correlating with 2-nonenal and 2-hexanone emission in the compatible interaction at any time.

Moreover, statistically significant differences between the log(ratio) of *TomloxC* induction were measured at 7 dpi in the resistant MOV with respect to the susceptible MOM (Figure 5A), in agreement with the further over-emission of (*E*)-2-pentenal during ETI establishment at 14 dpi (Figure 4), while no differences were found between both interactions at 3 dpi. Interestingly, the significantly higher log(ratio) of *TomloxD* (Figure 5C) and *AOS* (Figure 5F) in MOV at 3 dpi could explain the levels of JA accumulation at 3 dpi (Figure 3B), as well as the overexpression of *JAZ7* (Figure 2B) in this resistant isogenic line. Additionally, the differential terpene emission in the MOV immune plants was in accordance with the statistically significant higher levels of *MTS1* analyzed at 3 dpi and 7 dpi (Figure 5G). A repression in both lines and times was observed for *SAMT1* gene, implicated in SA methylation, the downregulation being significantly lower in MOV than in MOM.

## 3. Discussion

Fusarium wilt is one of the most important diseases in tomato crops, which is caused by the soil-borne fungus *Fusarium oxysporum* f. sp. *lycopersici* [2]. Nowadays, neither fungicide treatments nor culture methods are proven to be effective. The discovery and subsequent introgressive hybridization of resistance genes (*I*) in tomato plants has resulted in better crop development as well as yield improvement [6]. Hence, the main goal of this study was to explore the volatile chemical composition and the main defensive phytohormones involved in the resistant Movione (MOV) and susceptible Momor (MOM) isogenic lines of Moneymaker tomato plants, carrying or not carrying the *I* resistance gene, upon fungal infection. Specifically, the identification of the VOCs emitted by tomato leaves after ETI establishment could provide us with new resistance inducers for the treatment of fusarium wilt.

To characterize both compatible and incompatible interactions, the gene activation of some defensive routes together with the levels of different signal molecules, e.g., SA, JA, and ABA, were measured at several time points (Figure 2 and Figure 3). The analysis of SA accumulation showed higher SA levels in susceptible *Fol*-infected MOM plants when compared to resistant MOV plants (Figure 3A), which is in accordance with higher *PR1* expression observed in these isogenic lines (Figure 2A). In agreement with our results, accumulation of SA in virulent infections, such as those produced by *Fol*, CEVd, tomato spotted wilt virus, *Xanthomonas campestris,* or *Pseudomonas syringae,* has also been described in tomato plants [11,14,24,25]. An SA signaling positive regulation was described in the compatible interaction between *Fusarium oxysporum* and *Arabidopsis thaliana* [26], which was confirmed by the hyper susceptibility of *NahG* transgenic plants unable to accumulate this hormone [27]. Besides, exogenous application of SA through root feeding and foliar spray induced resistance against *Fol* in tomatoes [28]. These results point to the defensive role of this phenolic compound in the tomato-*Fol* interaction. However, the endogenous accumulation of SA in virulent infections suggests that its presence is not sufficient to generate resistance. The low *PR1* expression with the minimal SA levels measured during the incompatible interaction suggests that the resistance of tomato plants to *Fol* is not mediated by SA, pointing out some antagonism with other defensive routes, such as the JA-mediated pathway [29].

In this sense, a statistically significant activation of both the JA marker gene *JAZ7* (Figure 2B), and the *TomloxD* and *AOS* genes involved in JA biosynthesis (Figure 5C,F) was found during the ETI establishment, indicating that tomato plants’ resistance to fusarium wilt could be mediated by this phytohormone at the onset of the infection process. In fact, JA perception, but not its biosynthesis, is critical to the fusarium wilt development in *Arabidopsis thaliana* [30]. Besides, jasmonate-deficient tomato mutants showed hypersusceptibility to the pathogen [31], and exogenous JA treatments in wheat reduced both the symptoms of fusarium wilt and mycelia growth [32]. Most recent studies suggest that host–*Fusarium* interaction is governed by JA, and that compromised JA levels are associated with increased susceptibility [33].

Generally, ET and JA are associated with plant resistance mechanisms against necrotrophic pathogens [12]. A higher induction of the *ACS2* gene was observed in susceptible MOM plants when compared to resistant MOV plants after fungal infection at all times analyzed (Figure 2C), showing a maximum at 14 dpi. Transcriptomic studies point out the ET-mediated activation of isolated genes during the first steps of the infection, prior to the signaling of other hormones such as JA, SA, or ABA [34]. All these results imply that the defensive tomato response against *Fol* appears to be firstly associated with ET/JA-mediated genes, but this response appears not to be effective enough to stop the fungal colonization [26]. Our results suggest an accompaniment of ET in the SA signaling during the compatible interaction. In *Arabidopsis,* an antagonism between SA and ET/JA routes against *Fusarium oxysporum* has been described [27]. Otherwise, ET has always been associated to symptoms [35] and senescence [36], therefore the *ACS2* induction in susceptible tomato plants could involve ET not only in the gel formation within the xylem vessels to avoid the access to the pathogen, but also in the symptom development [37].

ABA is usually related to abiotic stress, although some defensive role in biotic interactions has also been observed [38]. Particularly, the ABA-mediated resistance against fungal pathogen has been associated to callose deposition [39]. In our study, a significant *LEA* induction (Figure 2D), as well as an ABA accumulation at 7 dpi (Figure 3C), was measured in the susceptible MOM tomato plants. These results coincide with those obtained in *Arabidopsis,* in which ABA promotes *Fusarium oxysporum* susceptibility [40]. Besides, the occlusion of the xylem vessels during the compatible tomato–*Fol* interaction could resemble drought conditions, where a reduction of the transpiration rate and leaf expansion, as well as the ABA-mediated stomata closing, allow plants to survive [41].

All the above data indicate that the different accumulation patterns of these three signal molecules depend on the diversity of pathogens with a range of lifestyles. To our knowledge, this is the first study in which the levels of SA, JA, and ABA have been measured in two different isogenic tomato lines (susceptible and resistant) infected with *Fol*.

Interestingly, we also observed divergences in volatile chemical composition upon fungal infection. Specifically, the VOC emission of a diseased leaf was enriched in C9 and C6 fatty acid derivatives, while that of a resisting leaf was characterized mainly by terpenes and C5 fatty acid derivatives (Figure 4).

A significant production of oxidized compounds such as ketones and aldehydes, esters from the defensive SA response (MeSA, EtSA), and some compounds from fatty acid degradation (2-hexanone, 2-nonenal), were associated with susceptible tomato plants infected by *Fol* (Table S3 and Figure 4). The differential 2-nonenal and 2-hexanone emission was in agreement with a significant induction of the relative expression levels of *TomloxA* at 3 dpi (Figure 5B) and *TomloxF* and *HPL* at 7 dpi (Figure 5D,E), respectively. In contrast, VOCs involved in the early resistance of tomato MOV to *Fol* were some monoterpenes, apocarotenoids, and sesquiterpenes while some benzenoids (isobutylbenzene, methylbenzene, benzeneacetaldehyde) and fatty acid derivatives (2-ethylhexanoic acid, 5-ethyl-2-furanone, (*E*)-2-pentenal) were implicated in the late resistance (Table S2). The statistically significant induction of both *MTS1* at any time point along with *TomloxC* at 7 dpi matched with the specific monoterpene and C5 volatiles emitted in the ETI establishment.

Historically, VOCs have been associated with fruit quality and plant defense against herbivores. Recently, new plant–pathogen interactions have been studied to find novel biological properties of these compounds in the agrochemical industry. Compounds emitted that are related to phytophagous insects and wounding are mainly metabolites derived from fatty acid degradation and terpenoids [42,43].

Fatty acids and their derivatives have been well studied due to their antimicrobial properties [44]. Hexanoic acid inhibits *Fol* mycelia growth at high concentrations, and reduces its germination at low concentrations [45]. Its antifungal effect is due to the increase of the membrane hydrophobicity, destabilizing them and inhibiting the interaction with proteins and lipids in the cell surface [46]. Besides, emissions of the products of the *LOX* pathway, such as C6 aldehydes and alcohols and their derivatives, generally known as GLVs (green leaf volatiles) have been described in biotic stresses [47]. Specifically, GLVs are emitted by leaves undergoing direct damage and they are related to infection severity and the degradation of the cell wall in leaves [48]. Esters of GLVs have also been described to be emitted by tomato plants displaying ETI upon *Pseudomonas syringae* pv. *tomato* infection [14].

Terpenoids are emitted due to systemic damage associated with the number of and injury in the trichomes of tomato plants [49] and other secondary stresses [48]. Terpenoids like monoterpenes and sesquiterpenes have been studied in relation to insect–plant interactions for their toxicity and repellent properties [43]. By now, a few studies report that root pathogens like *Fol* induce a foliar emission of this type of compounds in their hosts as we observed in this study. β-caryophyllene was identified as a terpenoid which generated a plant-growth promotion effect in the interaction between lettuce and *Fusarium oxysporum* [50], and 3-carene was also identified as a VOC emitted by healthy tomato roots and infected ones by *Fol* [51].

Benzenoids are known because of their biological activities, and in tomato–*Pseudomonas* interaction, a higher concentration of phenolic compounds has been observed, provoking a 60% reduction in a subsequent *Fol* infection [52]. MeSA is a volatile molecule derived from SA which takes part in SAR [53]. This phenolic compound is able to travel across the phloem and in the air as a volatile signal among plants, producing SA to trigger the systemic defense in the host and the neighboring plants [42]. An accumulation of MeSA has also been identified in tomato plants infected with a virulent bacteria [14]. Besides, MeSA has been described to be involved in tomato defense response against *Fol*, since SAMT-silenced tomato are less susceptible to a virulent strain of this root-invading fungus [25]. The over-emission of MeSA observed in the susceptible MOM plants caused by *Fol* infection (Figure 4) is in accordance with the SA levels analyzed in these plants (Figure 3A), thus confirming that the endogenous presence of this phenolic defensive phytohormone and its methylated form cannot prevent the infection progress. Furthermore, our findings appear to indicate that MeSA levels correlate with SA accumulation, unlike *SAMT1* induction (Figure 5H), pointing out the importance of substrate content in the emission of this volatile.

The new and wide range of compounds identified in immune MOV tomato plants upon *Fol* infection after ETI establishment leads to new potential chemical strategies for crop protection against the fusarium wilt or other similar diseases. In this sense, exogenous treatments with compounds differentially emitted by tomato MOV plants resisting *Fol* infection (some terpenoids, benzenoids, and 2-ethylhexanoic acid; detailed in Figure 4 and Table S2) could lead to the activation of plant defense against this fungus, therefore uncovering new resistance inducers. Besides, transgenic plants over-emitting these VOCs by the constitutive activation of their biosynthetic pathways could represent a biotechnological tool for plant resistance.

## 4. Materials and Methods

### 4.1. Fungal Strain and Inoculum Preparation

The fungal strain *Fusarium oxysporum* f. sp. *lycopersici (Fol)* race 1 (ATCC 48112) was obtained from the Spanish Type Culture Collection (CECT, Universitat de València, Spain) and fungal inoculum was prepared as previously described [54]. The fungus was grown in a liquid sporulation medium (per litre of distilled H_2_O: sucrose, 60 g; KH_2_PO_4_, 1 g; NaNO_3_, 7 g; MgSO_4_·7H_2_O, 0.5 g; KCl, 0.5 g; tryptone, 3 g) for 72 h at 25 °C under continuous light and stirring (200 rpm). Then, the solution was filtered to remove the mycelium, and the spores were sedimented by centrifugation at 3600 rpm for 5 min. A suspension of purified spores was prepared at a concentration of 10^6^ spores/mL by using a hemocytometer.

### 4.2. Plant Material and Fungal Inoculation

Tomato cultivars Momor (MOM) and Movione (MOV), which are isogenic lines of Moneymaker tomato (*Solanum lycopersicum* L.), were acquired from the Tomato Genetics Resource Center, UC Davis (accessions LA2828 and LA3472, respectively). MOV plants contain the *I* gene which confers resistance to *Fol* race 1 resulting in an incompatible interaction, while MOM plants lack the *I* gene, thus being unable to recognize the fungus which results in a compatible interaction and the development of disease [8].

Seeds were surface sterilized with a 1:1 mixture of commercial sodium hypochlorite and distilled water and were placed in wet vermiculite and irrigated with Hoagland solution. A total of 36 MOM and 36 MOV plants were used for each experiment. Two-week-old plants were pulled up from the vermiculite and inoculated. Half of them were mock-inoculated with water and the rest were infected with *Fol* by immersing the injured roots of two-week-old plants in the fungal spore suspension for 5 min according to [4]. Then, tomato plants were transferred into hydroponic conditions in Hoagland solution and oxygen supply under controlled conditions with a 16 h photoperiod at 5000 lux, a temperature of 25 °C/20 °C, and a relative humidity of 70%. Hypocotyls and leaves were sampled independently after 3, 7, and 14 days post-inoculation (dpi), homogenized under liquid nitrogen, and stored frozen at −80 °C for later analysis.

MOM and MOV plants were visually inspected for the evaluation of symptoms, and the disease index [17] was scored from 72 biological replicates at 3, 7, and 14 dpi. This time course was selected since susceptible infected MOM plants totally collapsed at 14 dpi, therefore choosing regular intervals of one week (7 and 14 dpi) and half a week (3 dpi). Three independent experiments were performed and data from a representative one are shown.

### 4.3. DNA Extraction and Fungal Quantification in Planta

The level of fungal presence in the vascular tissues of tomato plants was evaluated by real-time quantitative PCR (qPCR) [55]. Total DNA was extracted by adding 500 µL of extraction buffer to a small amount of homogenized infected tomato hypocotyls in an Eppendorf tube, and then incubated for 30 min at 65 °C. A volume of 450 µL of chloroform-isoamyl alcohol (24:1) was added to the mix, vortexed and centrifuged at 13,000 rpm for 10 min. Once the organic phase was removed, one volume of isopropanol was added to the aqueous phase and centrifuged at 13,000 rpm for 10 min. DNA sediment was washed twice with 70% ethanol and centrifuged for 5 min at 13,000 rpm. Pellet was resuspended in 50 µL distilled water containing 2% RNase and quantified using a Nanodrop ND-1000 spectrophotometer (ThermoFisher Scientific, Waltham, MA, USA).

For fungal quantification, qPCR analysis of DNA from infected hypocotyl tissues corresponding to 2 independent plants (3 technical replicates) were performed [55] using tomato actin as an endogenous reference gene. The PCR primers used to amplify the rDNA -intergenic spacer of *Fol* (rDNA-IGS, Genbank accession AB106019) were the following: 5′-GCTGGCGGATCTGACACTGT-3′ as the forward primer (sp1-2f) and 5′-CCTAAACCACATATCTCGTCCAAA-3′ as reverse primer (sp1-2r), according to [56]. For actin amplification (Genbank accession AB199316), 5′-CTAGGCTGGGTTCGCAGGAGATGATGC-3′ and 5′-GTCTTTTTGACCCATACCCACCATCACAC-3′ were used as the forward and the reverse primers, respectively.

### 4.4. RNA Extraction and RT-qPCR Analysis

RNA extraction was carried out using the TRIzol reagent (Invitrogen, Carlsbad, CA, USA) following the manufacturer’s protocol. RNA was cleaned by precipitation with one volume of 6 M LiCl, leaving at 4 °C for 3 h. Subsequently, RNA was recovered after centrifugation at 12,000 rpm for 15 min at 4 °C, washed with 3 M LiCl, dissolved in DEPC water, and quantified using a Nanodrop ND-1000 spectrophotometer. Concentration was adjusted to 1 µg/µL and DNA was removed using the TURBO DNAse kit (Ambion, Austin, TX, USA) according to the manufacturer’s directions.

For the quantitative RT-PCR (RT-qPCR) analysis, 1 µg of total RNA was employed to obtain the corresponding cDNA target sequences using an oligo(dT)_18_ primer and the PrimeScript Reverse Transcriptase kit (Perfect Real Time, Takara Bio Inc., Otsu, Shiga, Japan), following the manufacturer’s protocol. RT-qPCR was carried out as previously described [57] in a 10 µL volume using MicroAmpFast 96-Well ReactionPlate (Applied Biosystems, ThermoFisher Scientific) and the PyroTaq EvaGreen qPCR Master Mix (CMB, Cultek, Spain) in a 7500 Fast Real Time PCR System (Life Technologies, Carlsbad, CA, USA). A housekeeping gene transcript, elongation factor 1 *α* (*eEF1α*), was used as the endogenous reference. The PCR primers are listed in Table 1. Three technical repetitions were performed per plant.

### 4.5. HS-SPME Extraction and GC-MS Analysis of Volatile Compounds

The volatile organic compounds (VOCs) were extracted from 0.1 g of ground tomato leaves (6 biological replicates) weighed in a 10 mL headspace screw-cap vial according to Rambla et al. 2015 [58]. One milliliter of saturated CaCl₂ at pH 6 and 100 µL EDTA 750 nM at pH 7.5 were added, and the mixture was sonicated for 5 min. Volatile compound extraction was performed by head space solid-phase microextraction (HS-SPME). Samples were incubated at 50 °C for 10 min, extracted at the same temperature for 20 min, and adsorbed in a 65 µm PDMS/DVB fiber (Supelco, Bellefonte, PA, USA). Solid phase microextraction of the adhered compounds was carried out for 1 min at 250 °C in splitless mode using a CombiPAL autosampler (CTC Analytics, Zwingen, Switzerland). Then, the fiber was cleaned by exposing it for 5 min at 250 °C in an SPME fiber conditioning station (CTC Analytics) to prevent cross-contamination.

Separation of the compounds was performed by using an Agilent 6890N gas chromatograph (Santa Clara, CA, USA) coupled to an Agilent 5975B mass spectrometer operating in electronic impact (EI) mode with 70 eV of ionization energy and 230 °C of source temperature. Chromatographic separation was carried out on a DB-5ms fused silica capillary column (60 m long, 0.25 mm i.d., 1 µm film thickness) using helium as the carrier gas at a constant flow of 1.2 mL/min. Temperature conditions established in the oven were 40 °C for 2 min, a ramp from 5 °C/min to 250 °C, and an isothermal at 250 °C for 5 min. Data acquisition was performed at 6 scans per second in an *m*/*z* range of 35–250. Chromatograms and mass spectra were acquired and processed using the Enhanced ChemStation F.01.03.2357 software (Agilent).

Unequivocal identification of the VOCs was performed by using commercial compounds served as standards. The coelution and the spectrum equivalence between the commercial compound and the one to determine confirmed unequivocally its identity. Other compounds were tentatively identified by comparing their mass spectra with those listed in the NIST 05 Mass Spectral library.

### 4.6. Salicylic, Jasmonic and Abscisic Acids Measurements

Aliquots (about 100 mg of fresh weight) of frozen tomato leaves (3 biological replicates) were extracted with 80% methanol −1% acetic acid. Deuterium-labeled hormones [^2^H_6_] ABA and [^2^H_4_] SA, were added as internal standards for ABA and SA quantification, whereas the compound dhJA (dihydrojasmonic acid) was used for JA quantification. For collecting the acid fraction containing SA, ABA, and JA, the extracts passed consecutively through HLB (reverse phase), MCX (cationic exchange), and WAX (ionic exchange) columns (Oasis 30 mg cartridges, Waters, Milford, MA, USA), as described in [59].

The final residue was dissolved in 5% acetonitrile −1% acetic acid, and the hormones were separated using a reverse phase (2.6 µm Accucore RP-MS column, 100 mm length x 2.1 mm i.d.) UPLC system (ThermoFisher Scientific) with a 5 to 40% acetonitrile gradient containing 0.05% acetic acid at 0.4 mL/min over 14 min. The hormones were analyzed by electrospray ionization and targeted-SIM using a Q-Exactive spectrometer (Orbitrap detector, ThermoFisher Scientific). The concentrations of hormones in the extracts were determined using embedded calibration curves and the Xcalibur 4.1 SP1 build 48 and TraceFinder 4.0 programs (ThermoFisher Scientific).

### 4.7. Statistical Analysis

Phenotypical data of MOM and MOV lines were referred to be comparative. Every parameter of the infected plants was divided into the average of their corresponding mock plants measurements making the data comparable between both lines.

The statistical analyses were done using the IBM SPSS Statistics v.23 package. To test the normality of the data a Kolmogorov–Smirnov was applied, and a *t*-test or a Mann–Whitney test were used for parametric and non-parametric data, respectively.

For the untargeted analysis of the volatile profile, the GC-MS data were processed with the MetAlign 041012 software (Wageningen University, Wageningen, Netherlands) for the alignment of the chromatograms and the quantification of each MS feature. The resulting dataset was submitted to a principal component analysis (PCA) study using the SIMCA-P software (v. 11.0, Umetrics, Umeå, Sweden) using unit variance (UV) scaling.

## 5. Conclusions

The compatible interaction between tomato susceptible plants and *Fol* was characterized by a long-lasting induction of genes involved in SA, ET, and ABA biosynthesis routes and a repression of the JA pathway. These results were in accordance with the accumulation of the SA and ABA hormones and the emission of volatile organic compounds like methyl- and ethyl-salicylate. Moreover, the disease index correlated with the production of volatile compounds derived from the oxidation of fatty acids.

The incompatible interaction between tomato plants harboring the *I* resistance gene and *Fol* was associated with an early activation of the JA route. Additionally, terpenoids like *p*-menth-1-en-9-al, 3-carene, elemene, geranylacetone or *α*-caryophyllene, some benzenoids, and 2-ethylhexanoic acid were produced during the ETI establishment. Some of these volatiles emitted by immune tomato plants could be employed as new resistance inducers for fusarium wilt treatment.

## Figures and Tables

**Table 1:** Primer sequences used for real-time quantitative PCR.

Gene	Forward Primer (5′-3′)	Reverse Primer (5′-3′)
*PR1*	ACTCAAGTAGTCTGGCGCAACTCA	AGTAAGGACGTTGTCCGATCGAGT
*ACS2*	GATGGATTTGCGTCCACTTT	GATCCAGGCGAGACGTTAG
*LEA*	AGCAGATGTTGGAAAAGGAGC	ATGCCTATGGTGGGGTATTGT
*TomloxA*	AAGAAAGCTGGAGTTTGAATGAA	TTGAAACTTTTCAGCTGGAATTA
*TomloxC*	GCAATGCATCATGTGC	GTAAATGTCGAATTCCCTTCG
*TomloxD*	GGCTTCGTTTACTCTCTGGCT	AAATCAAAGCGCCAGTTCTT
*TomloxF*	CCGAATCAAAGGGTGACTTT	GGTCTGTGATGATCGATTGC
*HPL*	AGCTACGGATTGCCGTTAGT	CCATTCTCTTGGTGAAGAA
*AOS*	CCTCTTCCTTCTCTTCACCAAA	GCCGGGTATAGTCCTGGTAGA
*MTS1*	TGGTGGTCACCTTCAAGAGA	GCCTTGTGGTGGAAATAGGA
*SAMT1*	TCCCAGAAACATTATACATTGCTGAT	AATGACCTAACAAGTTCTGATACCACTAA
*EF1α*	CCACCTCGAGATCCTAAGG	ACCCTCACGTATGCTTCCAG

## Data Availability

Data sharing is not applicable to this article.

## Supplementary Materials

The following are available online, Figure S1: *Fol* quantification in hypocotyl of infected Momor (MOM; white) and Movione (MOV; gray) tomato plants at 7 and 14 dpi by qPCR. Values obtained for the fungal rDNA IGS region (Genbank accession AB106019) were normalized in relation to actin gene (accession AB199316). DNA expression levels were expressed as the average values of a representative experiment. Asterisks point out statistical differences between MOM and MOV plants according to *t*-test with *p* < 0.001 (***). Figure S2: Score plot of the PCA based on the whole array of the mass spectra within an *m*/*z* range from 35 to 250. VOCs were analyzed from 6 independent leaves of mock inoculated Momor (MOM; white) and Movione (MOV; gray) tomato plants at 7 and 14 dpi. PC1 and PC2 explain the 17% and the 12% of variance separating the samples according to the timing and isogenic line, respectively. Table S1: Differences in physiological parameters observed in susceptible (MOM) and resistant (MOV) tomato plants at 14 days after *Fol* inoculation. Average ratios for *Fol*- and mock-infected plants corresponding to three independent experiments are shown ± standard deviation. Asterisks (*) point out the significant differences between MOM and MOV according to t-test with *p* < 0.05 (*) and *p* < 0.01 (**). Table S2: GC-MS-detected VOCs differentially emitted during ETI establishment in resistant MOV tomato plants infected by *Fol* at 7 and 14 dpi. The data are expressed as MOV/MOM ratio and the statistical analysis was done by a *t*-test between six biological replicates. The asterisk (*) indicates a tentative identification by comparison with the NIST library. ^A^ monoterpene, ^B^ sesquiterpene, and ^C^ nor-isoprenoid. In bold, statistically significant VOCs over-emitted at 7 and 14 dpi in resistant MOV plants upon *Fol* inoculation. Table S3: GC-MS-detected VOCs differentially emitted in susceptible MOM tomato plants infected by *Fol* at 7 and 14 dpi. The data are shown as MOV/MOM ratio and the statistical analysis was done by a *t*-test between six biological replicates. In bold, statistically significant VOCs over-emitted at both 7 and 14 dpi in susceptible MOM plants upon *Fol* inoculation.

## Sample Availability

Samples of the compounds are not available from the authors.
