# Supplementary material for: Signaling in the Tomato Immunity against Fusarium oxysporum
Source: Molecules. 2021 Mar 24;26(7):1818. doi: 10.3390/molecules26071818 (PMC8036676; doi:10.3390/molecules26071818)
Supplement: Supplementary file 1 [file molecules-26-01818-s001.pdf]

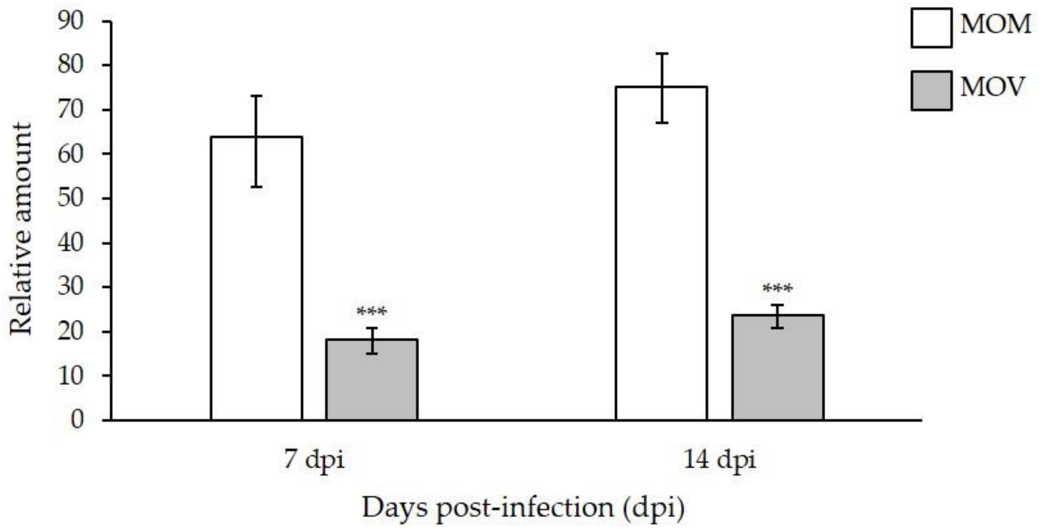

Figure S1. *Fol* quantification in hypocotyl of infected Momor (MOM; white) and Movione (MOV; gray) tomato plants at 7 and 14 dpi by qPCR. Values obtained for the fungal rDNA IGS region (Genbank accession AB106019) were normalized in relation to actin gene (accession AB199316). DNA expression levels were expressed as the average values of a representative experiment. Asterisks point out statistical differences between MOM and MOV plants according to *t*-test with $p < 0.001$ (***).

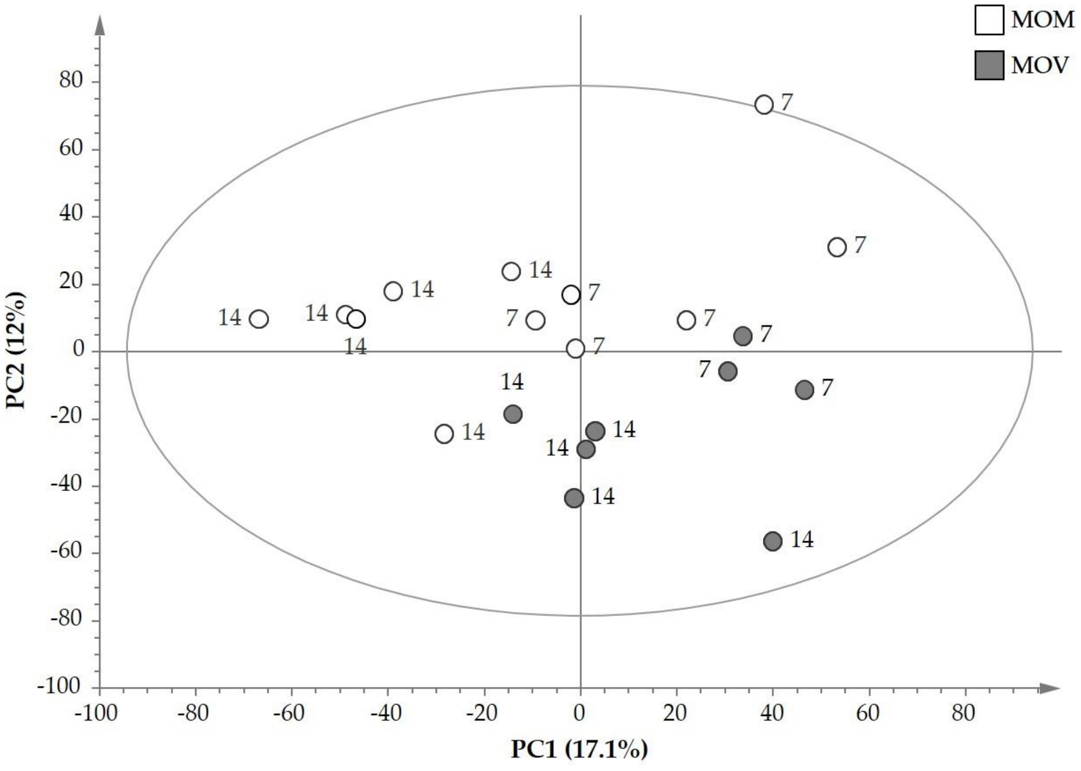

Figure S2. Score plot of the PCA based on the whole array of the mass spectra within an *m*/*z* range from 35 to 250. VOCs were analyzed from 6 independent leaves of mock inoculated Momor (MOM; white) and Movione (MOV; gray) tomato plants at 7 and 14 dpi. PC1 and PC2 explain the 17% and the 12% of variance separating the samples according to the timing and isogenic line, respectively.

Table S1. Differences in physiological parameters observed in susceptible (MOM) and resistant (MOV) tomato plants at 14 days after *Fol* inoculation. Average ratios for *Fol*- and mock-infected plants corresponding to three independent experiments are shown ± standard deviation. Asterisks (*) point out the significant differences between MOM and MOV according to *t*-test with $p < 0.05$ (*) and $p < 0.01$ (**).

Isogenic line	Height (*Fol*/Mock)	Hypocotyl Height (*Fol*/Mock)	Epicotyl Height (*Fol*/Mock)	Fresh Weight (*Fol*/Mock)	Water Intake (*Fol*/Mock)	Disease index
MOM	0.72 ± 0.21*	1.20 ± 0.33*	1.07 ± 0.60	0.55 ± 0.27*	0.74 ± 0.32	2.11 ± 1.13**
MOV	0.94 ± 0.21	1.00 ± 0.19	1.02 ± 0.20	0.84 ± 0.44	0.95 ± 0.49	0

Table S2. GC-MS-detected VOCs differentially emitted during ETI establishment in resistant MOV tomato plants infected by *Fol* at 7 and 14 dpi. The data are expressed as MOV/MOM ratio and the statistical analysis was done by a *t*-test between 6 biological replicates. The asterisk (*) indicates a tentative identification by comparison with the NIST library. [A] monoterpene, [B] sesquiterpene, and [C] nor-isoprenoid. In bold, statistically significant VOCs over-emitted at 7 and 14 dpi in resistant MOV plants upon *Fol* inoculation.

	COMPOUND	Rt (min)	ION	Ratio MOV / MOM 7 dpi	*p*-value	Ratio MOV / MOM 14 dpi	*p*-value
Fatty Acids Derivatives	2-ethylhexanoic acid *	26.84	88	1.800	0.027	0.976	0.987
	5-ethyl-2-furanone *	21.87	112	0.749	0.563	7.107	0.021
	(*E*)-2-pentenal	14.04	55	1.036	0.896	1.660	0.010
Terpenoids	*p*-menth-1-en-9-al *[A]	31.38	41	2.647	0.000	0.238	0.001
	2,6,6-trimethyl-1-cyclohexen-1-carboxaldehyde *[A]	31.43	41	1.093	0.002	0.783	0.179
	3-carene [A]	24.26	93	1.518	0.000	1.208	0.167
	(*Z*)-linalool oxide [A]	26.31	93	1.518	0.039	1.208	0.410
	beta-elemene *[B]	36.42	93	1.876	0.023	1.272	0.329
	unknown hydrocarbon sesquiterpene *[B] (unk 1)	37.99	105	1.602	0.038	1.271	0.220
	**alpha-caryophyllene [B]**	38.63	93	1.633	0.025	1.491	0.048
	gamma-elemene *[B]	34.85	121	1.554	0.039	1.024	0.827
	beta-caryophyllene [B]	37.69	93	1.454	0.148	1.413	0.050
	delta-elemene *[B]	34.73	121	1.703	0.017	1.073	0.689
	beta-ionone [C]	38.70	177	2.135	0.050	4.372	0.268
	dihydroactinidiolide *[C]	40.67	137	2.408	0.034	2.887	0.190
	geranylacetone *[C]	37.43	43	1.696	0.273	3.390	0.002
Benzenoids	isobutylbenzene *	28.24	91	2.268	0.143	10.491	0.000
	benzeneacetaldehyde *	25.40	91	1.158	0.603	3.083	0.018
	methylbenzene *	14.76	91	0.832	0.438	4.570	0.007
Others	2-ethyl-thiophene *	18.72	97	1.106	0.658	3.229	0.019
	**2,2-dimethyldecane ***	23.36	56	1.920	0.000	1.198	0.028
	**unknown nitrogen compound (unk 2)**	33.43	72	1.442	0.022	1.385	0.008
	unknown compound (unk 3)	37.53	173	2.868	0.032	1.066	0.731

Table S3. GC-MS-detected VOCs differentially emitted in susceptible MOM tomato plants infected by *Fol* at 7 and 14 dpi. The data are shown as MOV/MOM ratio and the statistical analysis was done by a t-test between 6 biological replicates. In bold, statistically significant VOCs over-emitted at both 7 and 14 dpi in susceptible MOM plants upon *Fol* inoculation.

	**COMPOUND**	**Rt (min)**	**ION**	**Ratio MOV / MOM 7 dpi**	***p*-value**	**Ratio MOV / MOM 14 dpi**	***p*-value**
Fatty Acids Derivatives	3-buten-2-one	7.89	55	0.253	0.039	4.971	0.049
	butanal	8.12	44	0.715	0.023	1.273	0.884
	2-butanone	8.16	43	0.553	0.000	1.073	0.153
	2-pentanone	11.30	43	0.860	0.121	0.842	0.006
	**2-hexanone**	15.29	43	0.766	0.004	0.873	0.030
	**3-heptanone**	19.13	57	0.825	0.004	0.879	0.046
	**2-heptanone**	19.27	43	0.867	0.026	0.875	0.017
	**2-nonenal**	29.01	83	0.328	0.019	0.496	0.037
Benzenoids	methyl salicylate	30.56	120	0.739	0.019	0.648	0.374
	ethyl salicylate	32.83	120	1.776	0.569	0.552	0.008
